# Effects of erythropoietin receptors and erythropoiesis-stimulating agents on disease progression in cancer

**DOI:** 10.1038/bjc.2012.42

**Published:** 2012-03-06

**Authors:** M Aapro, W Jelkmann, S N Constantinescu, B Leyland-Jones

**Affiliations:** 1 Institut Multidisciplinaire d’ Oncologie, Clinique de Genolier, Route du Muids 3, PO Box 100, Genolier CH-1272, Switzerland; 2 Institute of Physiology, University of Lübeck, Ratzeburger Allee 160, Lübeck D-23538, Germany; 3 Ludwig Institute for Cancer Research and de Duve Institute, Université Catholique de Louvain, Avenue Hippocrate 74, UCL 75-4, Brussels B-1200, Belgium; 4 Winship Cancer Institute, Emory University, School of Medicine, 1365C Clifton Rd NE, Ste 4014, Atlanta, GA 30322, USA

**Keywords:** disease progression, erythropoietin receptor, erythropoiesis-stimulating agents

## Abstract

Erythropoiesis-stimulating agents (ESAs) increase red blood cell (RBC) production in bone marrow by activating the erythropoietin receptor (EpoR) on erythrocytic-progenitor cells. Erythropoiesis-stimulating agents are approved in the United States and Europe for treating anaemia in cancer patients receiving chemotherapy based on randomised, placebo-controlled trials showing that ESAs reduce RBC transfusions. Erythropoiesis-stimulating agent-safety issues include thromboembolic events and concerns regarding whether ESAs increase disease progression and/or mortality in cancer patients. Several trials have reported an association between ESA use and increased disease progression and/or mortality, whereas other trials in the same tumour types have not provided similar findings. This review thoroughly examines available evidence regarding whether ESAs affect disease progression. Both clinical-trial data on ESAs and disease progression, and preclinical data on how ESAs could affect tumour growth are summarised. Preclinical topics include (i) whether tumour cells express EpoR and could be directly stimulated to grow by ESA exposure and (ii) whether endothelial cells express EpoR and could be stimulated by ESA exposure to undergo angiogenesis and indirectly promote tumour growth. Although assessment and definition of disease progression vary across studies, the current clinical data suggest that ESAs may have little effect on disease progression in chemotherapy patients, and preclinical data indicate a direct or indirect effect of ESAs on tumour growth is not strongly supported.

Anaemia is often associated with chemotherapy treatment because of the myelosuppressive effects of chemotherapy and/or the cancer disease itself (Groopman and Itri, 1999; Ludwig *et al*, 2004). As anaemia can lead to fatigue and decreased quality of life (Cella *et al*, 2004), its management is important for patient care. Anaemia therapies include red blood cell (RBC) transfusions and erythropoiesis-stimulating agents (ESAs), which increase RBC production in bone marrow by activating the erythropoietin receptor (EpoR) on erythrocytic-progenitor cells (Egrie *et al*, 1986, 2003). Transfusions quickly increase haemoglobin levels but are associated with risks such as transmission of infectious pathogens and transfusion-related acute-lung injury (Klein *et al*, 2007). Large, placebo-controlled clinical trials have shown that ESAs decrease transfusion rates in cancer patients (Littlewood *et al*, 2001; Vansteenkiste *et al*, 2002; Hedenus *et al*, 2003). Based on these trials, ESAs such as epoetin alfa and darbepoetin alfa are approved in the United States (Amgen, 2011; Centocor Ortho Biotech Products, 2011) and other countries (EMEA, 2011; eMC, 2011) for treating anaemia in patients with non-myeloid malignancies receiving chemotherapy. Additional ESAs are approved outside the United States for this indication (Jelkmann, 2010).

Clinical and preclinical research has examined the benefits and risks associated with ESA use. Although ESAs decrease transfusions, they are associated with an increase in thromboembolic events (Bennett *et al*, 2008; Glaspy *et al*, 2010). The potential for ESAs to affect disease progression and/or mortality in cancer patients has also been of concern (Bennett *et al*, 2008; Bohlius *et al*, 2009; Tonelli *et al*, 2009; Glaspy *et al*, 2010). To better understand ESA-related safety issues, several recent large meta-analyses have examined how ESAs affect thromboembolic events and mortality. Disease progression, however, was not always addressed (Bennett *et al*, 2008; Bohlius *et al*, 2009). Difficulties in analysing disease progression include variation in endpoints (e.g., progression-free survival, locoregional control, tumour response, etc.) and varying quality of disease-assessment measurements. Nonetheless, understanding if and how ESAs impact disease progression are key issues. This narrative review discusses clinical-trial data regarding ESAs and disease progression as well as preclinical research regarding how ESAs could affect disease progression at a cellular/molecular level.

## Erythropoiesis-stimulating agents and disease progression: evidence from clinical trials

As anaemia is an independent-risk factor for mortality in many cancer types (Caro *et al*, 2001), one question of interest was whether treating anaemia with ESAs improves cancer-patient survival. Higher haemoglobin levels were postulated to enhance tumour-tissue oxygenation, leading to increased chemotherapy and/or radiotherapy efficacy (Hadland and Longmore, 2009). Though some preclinical (Thews *et al*, 1998; Mittelman *et al*, 2001) and early clinical data (Littlewood *et al*, 2001; Vansteenkiste *et al*, 2002) suggested an ESA-associated survival benefit, other trials suggested that ESAs increased disease progression and/or mortality. Currently, the ESA-product labelling (Amgen, 2011; Centocor Ortho Biotech Products, 2011; EMEA, 2011; eMC, 2011) describes eight clinical trials of concern that suggest ESA use increases disease progression and/or mortality in cancer patients (Table 1). Two studies were performed in the non-indicated setting of radiotherapy treatment only (Henke *et al*, 2003; Overgaard *et al*, 2010), two in the non-indicated anaemia-of-cancer setting (patients received neither chemotherapy nor radiotherapy) (Wright *et al*, 2007; Smith *et al*, 2008) and four in the indicated chemotherapy setting (Hedenus *et al*, 2003; Leyland-Jones *et al*, 2005; Thomas *et al*, 2008; Untch *et al*, 2011b). As these eight studies are a focus for concerns regarding ESAs, they are described in more detail below according to their oncology setting.

### Radiotherapy only setting

The Erythropoietin in Head and Neck Cancer (ENHANCE) study was one of the first clinical trials to raise concerns about ESAs and disease progression (Table 1) (Henke *et al*, 2003). In this study, head and neck cancer patients scheduled to receive radiotherapy only (*N*=351) were randomised to placebo or epoetin beta 300IU kg^−1^ 3 × weekly. This study tested whether using ESAs to increase haemoglobin to ⩾14 g dl^−1^ would enhance curative radiation by improving tumour oxygenation. An intent-to-treat analysis stratified by cancer stage and treatment indicated that ESA-treated patients experienced increased locoregional progression (relative risk (RR)=1.69; 95% CI: 1.16–2.47; *P*=0.007) and decreased survival (RR=1.39; 95% CI: 1.05–1.84; *P*=0.02). However, results analysed ‘per protocol’ indicated no significant effect of ESAs on disease progression. Study limitations included multiple protocol violations and imbalances in some baseline characteristics (e.g., more ESA-treated patients had relapsed cancer and were smokers). A *post-hoc* analysis of EpoR expression in tumour cells from ESA-treated and control patients suggested that locoregional progression-free survival was poorer in ESA-treated patients with EpoR-positive tumours (Henke *et al*, 2006). However, the EpoR antibody used was later shown to be non-specific because of cross-reactivity with heat-shock proteins (Elliott *et al*, 2006; Brown *et al*, 2007).

The Danish Head and Neck Cancer-10 (DAHANCA-10) study in head and neck cancer patients receiving radiotherapy only evaluated whether using darbepoetin alfa (150 *μ*g weekly) to maintain haemoglobin between 14.5 and 15.5 g dl^−1^ could improve the effect of primary-curative radiotherapy (Overgaard *et al*, 2010). Study outcomes were recently reported (Overgaard *et al*, 2010) but have yet to be published in a manuscript. Overall, results from 514 patients showed poorer disease progression and survival outcomes in the darbepoetin arm. The RR was 1.51 (95% CI: 1.05–2.17) for 5-year locoregional control, 1.52 (95% CI: 1.07–2.16) for disease-free survival, and 1.39 (95% CI: 0.98–1.97) for overall survival (Overgaard *et al*, 2010).

Although the ENHANCE and DAHANCA-10 trials suggested ESA use increases disease progression, this finding was not replicated in two randomised, controlled trials in the radiotherapy setting for the treatment of patients with head and neck cancer (Table 1). The Radiation Therapy Oncology Group (RTOG 99-03) trial that evaluated 40 000 IU weekly erythropoietin (Epo) to maintain haemoglobin between 9.0 and 13.5 g dl^−1^ (Machtay *et al*, 2007) and the controlled EPO-GBR-7 trial (Hoskin *et al*, 2009) that evaluated 10 000 IU three times weekly epoetin alfa (haemoglobin <12.5 g/dl) or 4000 IU three times weekly epoetin alfa (haemoglobin ⩾12.5 g dl^−1^), did not show ESA use increased disease progression. Nonetheless, based on the ENHANCE and DAHANCA-10 studies, the ESA-product labelling does not recommend ESA use in the radiotherapy-only setting.

### Anaemia of cancer setting

The EPO-CAN-20 study evaluated non-small cell lung cancer patients randomised to epoetin alfa (40 000 IU weekly) or placebo (Wright *et al*, 2007). Enrolled patients received neither chemotherapy nor radiotherapy, although this was not stipulated in the trial design. An unplanned-interim analysis (*N*=66) indicated that ESA use increased mortality (hazard ratio (HR)=1.84; 95% CI: 1.01–3.35; *P*=0.04). Though the target study size was 300 patients, concerns about ESA-associated mortality led to study termination after 70 patients were randomised. Final results indicated that death occurred in 32 out of 33 patients receiving ESA and in 34 out of 37 patients receiving placebo. Kaplan–Meier curves of overall survival indicated that the median time to death favored placebo treatment (131 days) compared with ESA (68 days; *P*=0.04). Disease progression data were not formally collected.

The AMG 20010103 study evaluated 985 patients with non-myeloid malignancies and anaemia of cancer randomised to receive darbepoetin alfa (6.75 *μ*g kg^−1^ every 4 weeks) or placebo (Smith *et al*, 2008). No disease progression data were collected per study protocol, but a mortality analysis adjusted for stratification factors that impact ESA response indicated increased mortality in the ESA arm (HR=1.22; 95% CI: 1.03–1.45; *P*=0.022) (Smith *et al*, 2008). However, exploratory analyses (adjusted for baseline imbalances or known prognostic factors and for stratification factors that impact ESA response) diminished the mortality HR and statistical significance (HR=1.15; 95% CI: 0.97–1.37; *P*=0.121), suggesting that the possible negative effect of ESAs did not apply to all patient subsets. Of note, the mortality HR (95% CI) was 0.95 (0.73–1.23) for female patients compared with 1.32 (1.05–1.66) for male patients; however, the interaction between sex and treatment group was not statistically significant (*P*=0.066) (Smith *et al*, 2008).

Based on mortality data from the EPO-CAN-20 and AMG 20010103 studies, the ESA-product labelling does not recommend ESA use in the anaemia-of-cancer setting.

### Chemotherapy setting

The Breast Cancer Erythropoietin Survival Trial (BEST) was one of the first chemotherapy studies to report an association between increased mortality and ESA use (Leyland-Jones *et al*, 2005). Patients (*N*=939) with metastatic breast cancer were randomised to either epoetin alfa (40 000 IU weekly) or placebo as needed for up to 12 months; haemoglobin levels were maintained between 12 and 14 g dl^−1^. Although the target sample size was achieved, an independent data-monitoring committee recommended early termination of study-drug administration because of an interim analysis that indicated higher mortality in the ESA arm. In an intent-to-treat analysis adjusted for demographic and prognostic factors, mortality was reported as significantly higher in the ESA arm at 12 months (HR=1.36; 95% CI: 1.053–1.753; *P*=0.02). However, no difference in progression-free survival was observed (HR=1.00; *P*=0.98). An article written on behalf of the BEST investigators suggested that study-design issues (including possible imbalances in risk factors between study arms) (Leyland-Jones, 2003) may have prevented a conclusive interpretation of trial results. In addition, understanding the disease progression results may have been hampered by lack of prespecified tumour assessments at study entry, during the study, and during follow-up (Johnson and Johnson Pharmaceutical Research and Development LLC, 2004).

The AMG 20000161 study evaluated patients with lymphoproliferative malignancies receiving chemotherapy (*N*=344), who were randomised to receive darbepoetin alfa (2.25 *μ*g kg^−1^ weekly) or placebo for 12 weeks. The target haemoglobin level was 13–14 g dl^−1^ for women and 13–15 g dl^−1^ for men (Hedenus *et al*, 2003). The protocol was amended to allow collection of long-term follow-up data for survival and disease progression. Hedenus *et al* (2003) reported that after a median follow-up of 11 months, an initial analysis of long-term data indicated no difference between treatment groups for disease progression or death. After a median follow-up of 29 months, a prespecified analysis indicated higher mortality rates in ESA-treated patients (HR=1.36; 95% CI: 1.02–1.82) (Amgen, 2011). Erythropoiesis-stimulating agent use was not reported to affect disease progression (Amgen, 2011). Only limited conclusions can be drawn, however, as this study was neither designed to evaluate long-term survival or disease progression outcomes nor stratified to balance relevant prognostic factors.

The Gynaecologic Oncology Group (GOG)-191 study was conducted in cervical cancer patients receiving chemoradiotherapy who were randomised to receive or not receive recombinant Epo (40 000 IU weekly) during treatment (the overall radiation treatment time was ⩽8 weeks). This trial assessed whether maintaining haemoglobin levels of 13–14 g dl^−1^ would improve survival and progression outcomes (Thomas *et al*, 2008). Based on concerns of increased thromboembolic events in the ESA arm, the study closed after <25% of the planned accrual (460 patients were targeted to allow for 165 recurrences within 2 years). After a median follow-up of 37 months, results from 109 patients (52 in the control arm and 57 in the ESA arm) indicated that 25% of control patients and 33.3% of ESA-treated patients experienced disease recurrences; however, this result was not statistically significant (*P*=0.65) (Thomas *et al*, 2008). At ∼3 years, 73% of control patients and 61% of ESA-treated patients were still alive; 65% of control patients and 60% of ESA-treated patients were progression-free (Thomas *et al*, 2008). As this trial closed prematurely, the effect of ESAs on progression and mortality in this study remains undetermined.

The Preoperative Epirubicin Paclitaxel Aranesp (PREPARE) study evaluated the effect of preoperative dose-dense, dose-intensified chemotherapy with anthracyclines and taxanes in breast cancer patients (*N*=733). A second randomisation assigned patients to receive or not receive darbepoetin alfa 4.5 *μ*g kg^−1^ every 2 weeks to maintain haemoglobin concentrations between 12.5–13 g dl^−1^. Secondary endpoints included the effect of darbepoetin alfa on disease-free survival and overall survival. After a median follow-up of ∼3 years, an unplanned-interim analysis of 733 patients indicated that survival and progression-free survival rates were lower in ESA-treated patients (this difference was not statistically significant) (Amgen, 2008). Final results from the PREPARE trial were recently published in two manuscripts (Untch *et al*, 2011a, 2011b). When comparing ESA-treated patients with control patients, the 3-year estimated HR (95% CI) was 1.31 (0.99–1.74; *P*=0.061) for disease-free survival and 1.33 (0.91–1.95; *P*=0.139) for overall survival (Untch *et al*, 2011b). Though these results suggest a trend of decreased disease-free survival with darbepoetin alfa use, the findings were not statistically significant. Darbepoetin alfa use did not affect pathological-complete response (Untch *et al*, 2011a).

In summary, of the eight oncology studies of concern described in the ESA-product labelling, the two conducted in head and neck cancer patients receiving radiotherapy only showed the strongest evidence for an association between ESA use and disease progression. A recent study level meta-analysis by Glaspy *et al* (2010) reported an odds ratio (OR) for disease progression for each of the eight studies of concern. These results also suggested that only the ENHANCE and DAHANCA-10 studies demonstrated a statistically significant impact of ESA use on disease progression (Table 1).

### Additional chemotherapy studies

As the eight studies of concern were added to the ESA-product labelling, several large trials published between 2008 and 2010 have reported data regarding ESAs and disease progression in the chemotherapy setting. Four larger additional studies are described below.

The randomised, placebo-controlled AMG 20010145 study in small-cell lung cancer patients receiving chemotherapy (*N*=596 evaluated) compared overall survival (primary endpoint) and disease progression (additional efficacy endpoint) in patients receiving darbepoetin alfa or placebo (haemoglobin <13 g dl^−1^) (Pirker *et al*, 2008). This is one of the few controlled ESA trials in which all patients received the same chemotherapy regimen and in which tumour progression was assessed radiographically using blinded-centralised review. Published results of analyses stratified by randomisation factors indicated no significant difference between the two arms for progression-free survival (HR=1.02; 95% CI: 0.86–1.21; *P*=0.82) or overall survival (HR=0.93; 95% CI: 0.78–1.11; *P*=0.43) (Pirker *et al*, 2008).

Results from an Arbeitsgemeinschaft Gynäkologische Onkologie (AGO) phase 3 trial (Moebus *et al*, 2010) were recently published. This trial compared dose-dense chemotherapy *vs* conventionally scheduled chemotherapy in high-risk primary breast cancer patients (stage II–IIIA with ⩾4 positive axillary lymph nodes). Patients in the dose-dense arm (*N*=641 evaluated) were additionally randomised to receive or not receive epoetin alfa (haemoglobin at 12.5–13 g dl^−1^). In *ad-hoc* analyses, the manuscript reported that epoetin alfa did not affect overall survival or event-free survival (defined as locoregional or distant relapse, contralateral breast cancer, second primary-cancer occurrence, or death) but that detailed information will be communicated in a later publication (Moebus *et al*, 2010). (Of note, the most recent available data from the AGO trial were used in the meta-analysis of controlled ESA trials by Glaspy *et al* (2010) that examined the impact of ESAs on mortality/disease progression).

Results from the large GHSG HD15EPO trial were also recently published. Patients (*N*=1328 evaluated for safety) with advanced Hodgkin's lymphoma receiving chemotherapy were randomised to epoetin alfa or placebo (haemoglobin at 12–14 g dl^−1^ during chemotherapy and <12 g dl^−1^ after chemotherapy) (Engert *et al*, 2010). Results indicated that after a median-observation period of 3 years, epoetin alfa had no impact on freedom-from-treatment failure (HR=0.87; 95% CI: 0.63–1.20) or overall survival (HR=0.74; 95% CI: 0.45–1.22) (Engert *et al*, 2010).

A LNH03-6B Groupe d’ Etude des Lymphomes de l’ Adulte (GELA) study is currently being conducted in patients with large B-cell lymphoma receiving chemotherapy (R-CHOP) (Delarue *et al*, 2011). Patients were secondarily randomised to darbepoetin alfa (*N*=238; initially to maintain haemoglobin at 13–15 g dl^−1^ and later amended to 13–14 g dl^−1^) or to receive best-supportive care (ESA and transfusions administered according to usual practices; *N*=362). A second interim analysis was recently reported and indicated that 3-year progression-free survival was 66% in the darbepoetin alfa arm and 58% in the control arm (HR=0.77; 95% CI: 0.59–0.99). In an exploratory analysis comparing patients treated with or without ESAs (40% of controls received ESAs as supportive care), the HR for progression-free survival was 0.73 (95% CI: 0.57–0.94) (Delarue *et al*, 2011).

Recent randomised, controlled ESA studies also suggest no significant impact of ESAs on disease progression in chemotherapy patients (Wagner *et al*, 2004; Reed *et al*, 2005; Bohlius *et al*, 2009; Gupta *et al*, 2009; Ludwig *et al*, 2009; Nagel *et al*, 2011). Of note, a retrospective study in 323 multiple myeloma patients receiving chemotherapy reported that median progression-free survival was significantly shorter (*P*<0.001) in ESA-treated patients compared with non-ESA-treated patients (Katodritou *et al*, 2008). In contrast, a retrospective study (Hershman *et al*, 2009) of chemotherapy patient data from the SEER-Medicare database (from 1991 to 2002) indicated that overall survival was similar between patients receiving ESAs (*N*=12 522) or not receiving ESAs (*N*=34 820). In recently reported preliminary final results, the ARA Plus study (*N=*1234) prospectively evaluated event-free survival and overall survival in a randomised controlled study of adjuvant chemotherapy with or without darbepoetin in node-positive breast cancer patients (Nitz *et al*, 2011). After a median follow-up of 40 months, there were no significant differences in 3-year event-free survival (89.2% *vs* 87.6%, *P*=0.97) or overall survival (95.4% *vs* 95.1%, *P*=0.85) between patients receiving darbepoetin *vs* standard of care, respectively.

### Meta-analyses of ESA trials

Several recent meta-analyses have examined ESA use and safety outcomes in cancer patients. The large meta-analyses by Bennett *et al* (2008) and Bohlius *et al* (2009) reported a negative ESA impact risk on mortality but not on how ESAs affect disease progression. A recent meta-analysis by Tonelli *et al* (2009) analysed 52 controlled ESA-oncology trials; this meta-analysis was unique in that it did not include the BEST trial (Leyland-Jones *et al*, 2005) and included studies examining preoperative-ESA therapy. This meta-analysis did summarise two trials (*N*=247) that reported numbers of complete and partial tumour responses. These numbers did not differ significantly between ESA-treated and control patients (risk ratio for complete response=0.88; 95% CI: 0.69–1.12; risk ratio for partial response=0.70; 95% CI: 0.44–1.11).

Table 2 lists six meta-analyses that examined disease progression data from more than two controlled-ESA studies. These six meta-analyses examine overlapping data as they include subsets of the same studies. The publications by Hedenus *et al* (2005), Boogaerts *et al* (2006), and Seidenfeld *et al* (2006) reported results from three smaller meta-analyses (<1200 patients each). These meta-analyses suggested no significant impact of ESAs on disease progression (Table 2) (Hedenus *et al*, 2005; Boogaerts *et al*, 2006; Seidenfeld *et al*, 2006). The larger meta-analysis by Ludwig *et al* (2009) described a patient-level analysis of six randomised, controlled darbepoetin alfa trials performed in chemotherapy patients (*N*=2122). Analyses stratified by study indicated that darbepoetin alfa had no effect on disease progression (HR=0.92; 95% CI: 0.82–1.03), progression-free survival (HR=0.93; 95% CI: 0.84–1.04), or mortality (HR=0.97; 95% CI: 0.85–1.1). Aapro *et al* (2009b) described a meta-analysis using individual patient-level data from 12 randomised, controlled epoetin beta studies (*N*=2297) conducted in the oncology settings of chemotherapy, radiotherapy only, and surgery. An un-stratified analysis indicated a reduced risk of disease progression in the epoetin beta-treated patients (HR=0.85; 95% CI: 0.72–1.01). A study-level meta-analysis by Glaspy *et al* (2010) examined disease progression in 26 controlled-ESA studies (*N*=9646). These studies were a subset of 60 studies identified in a literature search for controlled-ESA trials that reported mortality data in the chemotherapy, radiotherapy only, and anaemia-of-cancer settings (Table 1) (Glaspy *et al*, 2010). Results indicated that ESA use did not significantly impact disease progression (OR=1.01; 95% CI: 0.90–1.14) (Table 2).

Based on the balance of evidence to date, the six meta-analyses described above do not support an effect of ESAs on disease progression. However, safety data from some individual, controlled trials suggest that ESAs might affect disease progression and/or mortality in certain cancer patient populations (head and neck cancer patients receiving radiotherapy only may be at particular risk). The need for additional research to understand whether and how ESAs affect tumour cell growth has stimulated much preclinical work in this field.

## ESAs and disease progression mechanisms: evidence from preclinical studies

To explain the conflicting clinical data, several mechanisms for disease progression have been postulated. The most widely studied is whether an Epo-specific receptor exists on tumour cells, endothelial cells, or other non-erythrocyte progenitor cells.

### The EpoR and tumour cells

**Figure 1:**
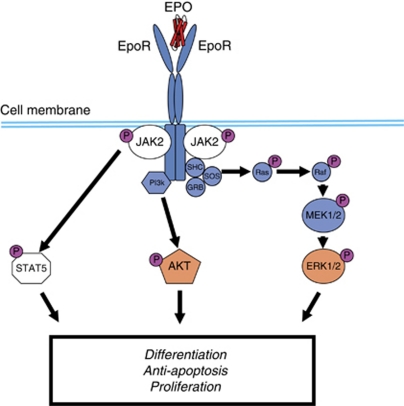
Schematic presentation of the signalling pathways activated by the EpoR on erythrocytic progenitor cells in response to Epo. When the surface of an erythrocytic progenitor cell is exposed to Epo, the pre-formed EpoR dimer undergoes a conformational change that stimulates autophosphorylation of JAK2 kinase, which is associated with the EpoR intracellular domains. In turn, JAK2 kinases phosphorylate tyrosine residues on the EpoR intracellular domains, which then serve as docking sites for various cytoplasmic signalling proteins such as the transcription factor STAT5 (signal transducer and activator of transcription 5). Stimulation of cytoplasmic signalling proteins such as STAT5, AKT, and ERK1/2 activates signalling cascades that can lead to cellular differentiation, anti-apoptotic effects, and cellular proliferation.

Like endogenous Epo, ESAs bind to and activate EpoR on erythrocytic progenitors (colony-forming units erythroid) in bone marrow (Broudy *et al*, 1991). This stimulates erythrocytic progenitor cells to proliferate and differentiate into RBCs. Without an ESA or endogenous Epo, erythrocytic precursors at the proerythroblast stage undergo apoptosis (Koury and Bondurant, 1988). Activation of EpoR stimulates JAK2 kinase, which binds to the cytosolic domains of the EpoR dimers (Figure 1) (Witthuhn *et al*, 1993). Activated JAK2 kinase stimulates multiple signalling pathways in erythrocytic precursor cells (Huang *et al*, 2001; Jelkmann *et al*, 2008). The JAK2 kinase is also an essential chaperone for translocating EpoR to the cell surface (Huang *et al*, 2001). It has been postulated that if tumour cells express EpoR, ESAs could activate these receptors to induce tumour cell proliferation (Hadland and Longmore, 2009). Thus, examining whether tumour cells express ESA-responsive EpoR has been of interest.

Several studies have suggested that tumour tissues and tumour cell lines express EpoR mRNA and also contain EpoR protein as demonstrated by western-blot analysis or immunohistochemistry (Sinclair *et al*, 2007; Jelkmann *et al*, 2008). However, technical issues have limited the validity of these findings and often qualitative, rather than quantitative, studies were performed. For example, studies examining EpoR-mRNA levels often used bulk tumour tissue, which can contain stromal cells and other cell types that infiltrate from blood. Moreover, several studies using western blot and/or immunohistochemistry may have yielded false-positive results because of use of commercially available polyclonal or monoclonal anti-EpoR antibodies later shown to lack specificity for EpoR (Elliott *et al*, 2006; Brown *et al*, 2007). In addition, many studies did not address whether EpoR was localised to the cell surface and/or whether it could be activated by an ESA. In a study suggesting that neuroblastoma SH-SY5Y cells contain EpoR molecules (<50 receptors per cell surface) that transmit an anti-apoptotic signal when exposed to an ESA (Um *et al*, 2007), cell surface EpoR could not be reliably detected using a radioactive Epo-binding assay.

Recent results have suggested that Epo can activate Jak2-mediated signalling and antagonise anti-HER2 (trastuzumab) therapy in breast cancer cells, and a non-significant decrease was observed in progression-free survival for patients treated with Epo and trastuzumab in a small, retrospective subgroup analysis (Liang *et al*, 2010). Another recent study also indicated partial reduction in the efficacy of cytotoxic therapy when combined with Epo in a mouse model of metastatic breast cancer (Hedley *et al*, 2011). However, interpretation of these studies is difficult because of the non-specific antibodies used to establish EpoR expression.

Although some studies have reported that hypoxia increases EpoR expression in non-haematopoietic cells (Farrell and Lee, 2004), research in breast carcinoma cell lines (LaMontagne *et al*, 2006) indicated that hypoxia did not affect EpoR expression and that Epo exposure did not induce cell proliferation or activate signalling molecules such as MAPK or Akt, which act downstream of EpoR. Studies in other tumour cell lines have also shown little/or no expression of EpoR protein and/or a lack of functional EpoR (Laugsch *et al*, 2008; Sinclair *et al*, 2008). Additional studies indicate that the *EpoR* gene is not amplified in tumour cells (Sinclair *et al*, 2008) and that Epo exposure does not induce tumour cell line proliferation or affect mortality in many animal tumour models (Osterborg *et al*, 2007; Sinclair *et al*, 2007).

Recently, a monoclonal antibody specific for EpoR was developed enabling detailed analysis of EpoR-protein expression and function (Elliott *et al*, 2010). Studies using this antibody have indicated that many tumour cell lines express low-to-undetectable levels of EpoR and that any EpoR present is not functional (exposure of the cell lines to Epo does not activate signalling molecules such as STAT5 that function downstream of EpoR) (Swift *et al*, 2010). In a study performed in primary human tumour samples from multiple epithelial tumour types, no cell surface or functional EpoR was detected (Rossi *et al*, 2009). These findings do not support the hypothesis that ESAs could increase the risk of disease progression by activating EpoR on tumour cells.

### Indirect mechanisms

Erythropoiesis-stimulating agent exposure could theoretically increase the risk of disease progression via indirect mechanisms. Intriguingly, recent findings suggest that activated monocyte/macrophage cells express EpoR and that binding of Epo to these cells can prevent NF-*κ*B activation, repress pro-inflammatory genes, and induce an immunosuppressive effect (Nairz *et al*, 2011). However, the possible immunomodulatory role of Epo influences on tumour growth is unknown. Tumour growth could also theoretically be influenced by changes in iron-dependent metabolism (especially in iron-deficient patients) (Shander *et al*, 2010) that result from an ESA-induced increase in RBC production. Research is needed to examine this possibility.

It has been proposed that ESAs could affect the cardiovascular system (van der Meer *et al*, 2004; Ribatti, 2010). There are reports showing *in vitro* angiogenic effects of Epo on human bone marrow-derived endothelial progenitor cells (EPCs) (Muller-Ehmsen *et al*, 2006; Zwezdaryk *et al*, 2007) and on endothelial cells derived from human adult myocardial tissue (Jaquet *et al*, 2002). Erythropoiesis-stimulating agent therapy has also been reported to increase circulating levels of EPCs (Bahlmann *et al*, 2003) and endogenous Epo levels were found to correlate with circulating EPCs in patients with ischaemic cardiomyopathy (Heeschen *et al*, 2003). However, ESA therapy did not affect the number of EPCs in donors for allogeneic peripheral blood stem cell transplantation (Kim *et al*, 2009) nor in patients with acute myocardial infarction (Taniguchi *et al*, 2010). In addition, long-term ESA treatment did not affect endothelial markers in patients on haemodialyses (Pawlak *et al*, 2007). At present, convincing evidence for an effect of ESAs on EPCs is missing in the clinical setting.

If blood vessel endothelial cells express EpoR, then ESA exposure could hypothetically stimulate neovascularisation or angiogenesis; blood vessel growth in a tumour could then enhance tumour proliferation. Some studies have suggested that endothelial cells contain EpoR mRNA and that Epo can stimulate endothelial cell proliferation (Anagnostou *et al*, 1990, 1994). However, a recent study demonstrated that human endothelial, renal, cardiac, and neuronal cells contain EpoR mRNA at levels 10–100-fold lower than those in cells highly responsive to Epo (Sinclair *et al*, 2010). In addition, low or no EpoR-protein expression was detected in these cell types using a recently developed specific anti-EpoR monoclonal antibody (Elliott *et al*, 2010). Erythropoiesis-stimulating agents were also observed to have no effect in a rat angiogenesis assay (Sinclair *et al*, 2010). These findings call into the question whether ESAs could indirectly stimulate disease progression via angiogenesis.

### Venous thromboembolic events

Venous thromboembolic events (VTEs) represent a known risk associated with ESA use in cancer patients (Bennett *et al*, 2008; Glaspy *et al*, 2010). This risk is described in the ESA-product labelling (Amgen, 2011; Centocor Ortho Biotech Products, 2011) and can be managed clinically (of note, a recent exploratory analysis of a controlled-ESA trial suggested that administering anti-thrombotic therapy with ESAs may lower VTE rates) (Aapro *et al*, 2009a). Although a link between VTEs and disease progression has not been established, it has been hypothesised that VTEs may account for the increased mortality associated with ESAs in some studies (Hadland and Longmore, 2009). One proposed hypothesis is that ESAs could increase the incidence of VTEs by stimulating platelet production. Although some results suggest that Epo binds to megakaryocytes (but not platelets) (Fraser *et al*, 1988), whether functional EpoR is expressed on megakaryocytes remains unclear (Grossi *et al*, 1989; Yonemura *et al*, 1992) and high levels of endogenous Epo do not appear to elevate platelet counts in humans (Akan *et al*, 2000). Studies evaluating platelet counts after ESA administration have reported varying results (Grossi *et al*, 1989; Yonemura *et al*, 1992; Ait-Oudhia *et al*, 2010). Furthermore, a clear association between increased platelet counts and an increased incidence of VTEs has not been demonstrated (Buss *et al*, 1994; Basser *et al*, 1997).

Another hypothesis is that as JAK2 kinase is a key mediator of EpoR activity (Figure 1), an association may exist between VTEs and JAK2 kinase activation. Research has shown that 30–50% of patients with splanchnic-vein thromboses associated with Budd-Chiari syndrome (including portal-venous and hepatic-vein thrombosis) harbour a somatic mutation of the *JAK2* gene (JAK2 V617F) that constitutively activates JAK2 kinase (Kiladjian *et al*, 2008). However, the JAK2V617F mutation is detected in multiple haematopoietic lineages (Ishii *et al*, 2006), and JAK2 kinase is essential for mediating signalling pathways for many cytokine receptors other than EpoR (Seidel *et al*, 2000). Therefore, no direct link between ESA-mediated JAK2 kinase activation and VTEs in cancer patients has been definitively established. Additional studies are required to understand the precise mechanism underlying the increased risk of VTEs associated with ESA use.

## Summary and conclusion

This review summarised results from clinical and preclinical studies that evaluated whether ESAs affect disease progression. Although there are important limitations on the quality and assessment of disease progression in these studies, the current meta-analyses suggest no overall effect of ESAs on disease progression. Several individual studies have shown a potential trend associating ESA use with increased disease progression. This suggests that ESAs may affect disease progression in particular cancer patient populations (e.g., head and neck cancer patients receiving radiotherapy only) and that additional research is needed to define these populations and how ESAs mediate this effect. Although indirect effects on tumours induced by increased RBC production are theoretically possible, preclinical data to date suggest that tumour cells either do not express EpoR or express low levels of EpoR molecules that are non-functional and/or are not present at the cell surface. Overall, the balance of current evidence does not support an effect of ESAs on either activating EpoR on tumour cells or indirectly stimulating disease progression via angiogenesis. Future clinical trials, meta-analyses, and preclinical research should provide additional data to guide evidence-based use of ESAs in cancer patients.

## Figures and Tables

**Table 1:** Controlled ESA oncology trials included in the meta-analysis by Glaspy *et al* (2010) that examined whether ESAs affect disease progression

**Study publication**	**Study number or alias**	**Tumour type**	**Number of patients analysed^a^**	**Odds ratio (95% CI) for disease progression^a^**
*Radiotherapy only setting*				
Henke *et al*, 2003^b^	ENHANCE	Head and neck	351	1.56 (1.01–2.39)
Overgaard *et al*, 2007^b^^,^^c^	SE-2002-9001 (DAHANCA-10)	Head and neck	513	1.77 (1.25–2.52)
Machtay *et al*, 2007	RTOG-99-03 PR99-03-046	Head and neck	148	1.05 (0.55–2.00)
Identified as unpublished in Glaspy *et al*, 2010^c^	EPO-GBR-7	Head and neck	300	1.02 (0.65–1.62)
				
*Anemia of cancer setting*				
Wright *et al*, 2007^b^	EPO-CAN-20	NSCLC	70	1.08 (0.30–3.95)^d^
Smith *et al*, 2008^b^	AMG 20010103	Non-myeloid malignancies	985	No disease progression data collected
				
*Chemotherapy setting*				
Osterborg *et al*, 1996^e^	MF4250	Haematological	144	1.20 (0.60–2.40)
Littlewood *et al*, 2001^e^	EPO-INT-10	Solid/non-myeloid malignancy	375	0.64 (0.40–1.02)
Pronzato *et al*, 2002^e^	EPO-INT-47	Breast	223	1.02 (0.46–2.26)
Vansteenkiste *et al*, 2002	AMG 980297	SCLC and NSCLC	314	0.58 (0.30–1.11)
Hedenus *et al*, 2003^b^	AMG 20000161	Haematological	344	1.08 (0.66–1.76)
Milroy *et al*, 2003^e^	EPO-INT-49	NSCLC	424	0.90 (0.57–1.41)
Blohmer *et al*, 2004^f^	AGO/NOGGO EPO-GER-8	Cervical	250	0.61 (0.33–1.13)
Vadhan-Raj *et al*, 2004^e^^,^^f^	PR00-03-006	Gastric and rectal	60	1.01 (0.35–2.94)
Chang *et al*, 2005^e^	EPO-CAN-17	Breast	354	0.82 (0.39–1.72)
Grote *et al*, 2005^e^	N93-004	SCLC	224	0.85 (0.50–1.44)
Leyland-Jones *et al*, 2005^b^	EPO-INT-76 (BEST)	Breast	939	0.84 (0.64–1.08)
Osterborg *et al*, 2005^e^	MF4467	Haematological	343	0.74 (0.44–1.25)
Witzig *et al*, 2005^e^	PR98-27-008	Mixed	344	1.20 (0.75–1.91)
Wilkinson *et al*, 2006^e^	EPO-INT-45	Ovarian	181	7.47 (0.95–58.54)
Engert *et al*, 2007^c^	GHSG HD15EPO	Hodgkin's lymphoma	688	0.86 (0.33–2.24)
Moebus *et al*, 2007 c	EPO-GER-7	Breast	643	1.05 (0.75–1.48)
Aapro *et al*, 2008	BRAVE	Breast	463	1.07 (0.82–1.40)
Pirker *et al*, 2008	AMG 20010145	SCLC	596	0.87 (0.52–1.46)
Strauss *et al*, 2008^f^	MARCH	Cervical	74	0.87 (0.32–2.33)
Thomas *et al*, 2008^b^^,^^f^	GOG-191	Cervical	109	1.02 (0.48–2.15)
Untch *et al*, 2008^b^^,^^c^	PREPARE	Breast	733	1.36 (0.97–1.91)

Abbreviations: BEST=Breast Cancer Erythropoietin Survival Trial; CI=confidence interval; DAHANCA-10=The Danish Head and Neck Cancer-10; ENHANCE=Erythropoietin in Head and Neck Cancer; Epo=erythropoietin; NSCLC=non-small cell lung cancer; PREPARE=The Preoperative Epirubicin Paclitaxel Aranesp; RTOG=The Radiation Therapy Oncology Group; SCLC=small cell lung cancer.

a Data are from the Glaspy *et al* (2010) study-level meta-analysis of controlled ESA trials in the oncology setting that reported survival data (these data are not from the ESA-product labels). Odds ratios were calculated using a random effects model. References listed refer to those used for the Glaspy *et al* (2010) meta-analysis.

b ESA oncology studies of concern described in the ESA-product labeling.

c As the Glaspy *et al* (2010) meta-analysis, updated publications have been made available for these studies.

d The study reported by Wright *et al* (2007) did not formally collect disease progression data. Disease progression was based on the reported deaths because of progressive lung cancer.

e Studies in which disease progression was evaluated only as part of tumour assessment.

f Patients received chemotherapy and radiotherapy.

**Table 2:** Summary of meta-analyses of controlled ESA-oncology trials that reported disease progression outcomes

**Meta-analysis publication^a^**	**Number of trials (number of patients)**	**Treatment setting**	**Disease progression statistic**
Hedenus *et al*, 2005	4 (1129)	4 chemotherapy	Hazard ratio for PFS=0.92 (95% CI: 0.78–1.07)
Boogaerts *et al*, 2006	3 (454)	3 chemotherapy	No risk identified with regard to ESA use and tumour progression
Seidenfeld *et al*, 2006	5 (688)	3 chemotherapy 2 radiotherapy only	Relative risk for complete response=1.00 (95% CI: 0.92–1.10)
Ludwig *et al*, 2009	6 (2122)	6 chemotherapy	Hazard ratio for disease progression=0.92 (95% CI: 0.82–1.03) Hazard ratio for PFS=0.93 (95% CI: 0.84–1.04)
Aapro *et al*, 2009b	12 (2297)	9 chemotherapy 2 surgery 1 radiotherapy only	Hazard ratio for disease progression=0.85 (95% CI: 0.72–1.01)
Glaspy *et al*, 2010	26 (9646)	21 chemotherapy 1 anemia of cancer 4 radiotherapy only	Odds ratio for disease progression=1.01 (95% CI: 0.90–1.14)

Abbreviations: CI=confidence interval; ESA=erythropoiesis-stimulating agent; PFS=progression-free survival.

a These meta-analyses examined >2 studies and included nearly the same studies or a subset of the same studies. Thus, they do not report independent effects based on analyses of completely different data sets.
